# Low mitochondrial DNA copy number induces chemotherapy resistance via epithelial-mesenchymal transition by DNA methylation in esophageal squamous cancer cells

**DOI:** 10.1186/s12967-022-03594-2

**Published:** 2022-08-29

**Authors:** Yuto Kubo, Koji Tanaka, Yasunori Masuike, Tsuyoshi Takahashi, Kotaro Yamashita, Tomoki Makino, Takuro Saito, Kazuyoshi Yamamoto, Tomoyuki Tsujimoto, Takashi Harino, Yukinori Kurokawa, Makoto Yamasaki, Kiyokazu Nakajima, Hidetoshi Eguchi, Yuichiro Doki

**Affiliations:** Department of Gastroenterological Surgery, Graduate School of Medicine, Osaka University, 2-2 E2 Yamadaoka, Suita, Osaka 565-0871 Japan

**Keywords:** Mitochondrial DNA, Epithelial-mesenchymal transition, Esophageal cancer, Chemotherapy, Mitochondrial membrane potential, DNA methylation

## Abstract

**Background:**

Esophageal squamous cell carcinoma (ESCC) is one of the most severe cancers and is characterized by chemotherapy resistance and poor prognosis associated with epithelial-mesenchymal transition (EMT). In a previous study, a low mitochondrial DNA (mtDNA) copy number was associated with poorer prognosis and induced EMT in ESCC. However, the detailed mechanism related to mtDNA copy number and EMT is unclear. The aim of this study was to clarify the mechanism by which a change in mtDNA copy number contributes to EMT and to examine treatment of chemotherapy resistance in ESCC.

**Methods:**

The association between low mtDNA copy number and chemotherapy resistance was investigated using specimens from 88 patients who underwent surgery after neoadjuvant chemotherapy. Then, the mtDNA content of human ESCC cell lines, TE8 and TE11, was depleted by knockdown of mitochondrial transcription factor A expression. The present study focused on modulation of mitochondrial membrane potential (MMP) and DNA methylation as the mechanisms by which mtDNA copy number affects EMT. mRNA and protein expression, chemotherapy sensitivity, proliferation, MMP and DNA methylation were evaluated, and in vitro and in vivo assays were conducted to clarify these mechanisms.

**Results:**

ESCC patients with decreased mtDNA copy number who underwent R0 resection after neoadjuvant chemotherapy had significantly worse pathological response and recurrence-free survival. Additionally, low mtDNA copy number was associated with resistance to chemotherapy in vitro and in vivo. mtDNA controlled MMP, and MMP depolarization induced EMT. Depletion of mtDNA and low MMP induced DNA methylation via a DNA methylation transcription factor (DNMT), and a DNMT inhibitor suppressed EMT and improved chemotherapy sensitivity in mtDNA-depleted ESCC cells, as shown by in vitro and in vivo assays.

**Conclusion:**

This study showed that decreased mtDNA copy number induced EMT via modulation of MMP and DNA methylation in ESCC. Therapeutic strategies increasing mtDNA copy number and DNMT inhibitors may be effective in preventing EMT and chemosensitivity resistance.

**Supplementary Information:**

The online version contains supplementary material available at 10.1186/s12967-022-03594-2.

## Background

An estimated 450,000 patients are diagnosed with esophageal cancer each year, with an estimated 400,000 deaths annually [1–3]. Esophageal cancer has the seventh highest incidence of cancers worldwide and the sixth worst prognosis due to its aggressiveness and poor survival rate according to global cancer statistics from 2018 [4]. The number of patients with esophageal cancer has been increasing over the past three decades [5]. Esophageal squamous cell carcinoma (ESCC) is the most common histological subtype of esophageal cancer, accounting for approximately 90% of all esophageal cancer cases worldwide [6].

The prognosis of advanced ESCC is poor even though neoadjuvant chemotherapy and chemoradiotherapy followed by surgery strategies have been developed and widely performed as effective treatments [7–9]. Therefore, new therapeutic targets for ESCC are required to improve the prognosis.

Mitochondria are eukaryotic intracellular organelles that have important cellular functions, such as metabolism, adenosine triphosphate (ATP) production (of which they do the majority) via oxidative phosphorylation (OXPHOS), reactive oxygen species (ROS) generation, intercellular signaling, and integration of apoptosis pathways [10, 11]. Each human cell contains hundreds to thousands of mitochondria, and mitochondrial DNA (mtDNA), which consists of a circular double-stranded structure with 16,569 base pairs and encodes 13 polypeptides that are essential for the assembly of respiratory enzyme complexes, is contained in mitochondria [12–15]. Decreased mtDNA copy numbers have been reported to be associated with tumor progression and poor prognosis of various cancers in the past few decades [16–19], and previous studies have shown that increased mtDNA copy numbers are associated with worse prognosis in several cancers, including gastrointestinal cancer [20–22]. As described above, the relationship between mtDNA copy number and prognosis varies by cancer type.

Our previous study showed that decreased mtDNA copy number was associated with significantly poorer overall survival and induced epithelial-mesenchymal transition (EMT) which had more invasive and migrate in ESCC [13]. EMT converts epithelial cells into mesenchymal cells, which have increased motility and changes in cell morphology and function that are associated with treatment resistance and tumor recurrence [23–25]. However, the mechanism underlying the association between mtDNA copy number and EMT is unclear.

Therefore, the aim of this study was to clarify the detailed mechanism by which mtDNA copy number changes contribute to EMT and to reveal strategies for overcoming treatment resistance in ESCC.

## Materials and methods

### Cell culture and establishment of depleted and increased mtDNA copy number

Human ESCC cell lines, TE8 (RBRC-RCB2098) and TE11 (RBRC-2100), were purchased from the RIKEN BioResource Center (Tsukuba, Ibaraki, Japan). These cells were cultured in RPMI-1640 medium supplemented with 10% fetal bovine serum (Thermo Fisher Scientific, Waltham, MA, USA), penicillin (100 IU/ml), and streptomycin (100 μg/ml) at 37 °C in a humidified incubator with 5% CO_2_. Depletion of mtDNA content was induced by knockdown of mitochondrial transcription factor A (TFAM) expression [26, 27]. Silencing of TFAM reduces mtDNA levels, since this factor is important for mtDNA packaging and maintenance [13, 28]. A short hairpin RNA designed by Sigma–Aldrich (St. Louis, MO, USA), MISSION TRC-Hs1.0, was applied for knockdown of TFAM expression in TE8 and TE11 cells. The depleted mtDNA cell lines “tfam-sh1” and “tfam-sh2” were established by targeting the following sequences of the TFAM gene: 5′-CGTCGCACAATAAAGAACAA-3′ (TRCN0000016094) and 5′-GCAGATTTAAAGAACAGCTAA-3′ (TRCN0000016097), respectively. In addition, the nontargeted sequence 5′-GGCGCGATAGCGCTAATAATTT-3′ (SHC016, Sigma–Aldrich, defined as ‘control-sh’) was used as the control for comparison (control-sh). The morphology of the cells was evaluated by optical microscopy (BZ-X710, KEYENCE, Osaka, Japan) [13]. In addition, an increased mtDNA copy number was established as follows: 1 × 10^6^ cells were plated in 1.25 ml of complete medium without antibiotics in 6-well plates and transfected with 7 mg of a plasmid containing the TFAM-encoding gene (OriGene, RC215488) using 12 μl of Lipofectamine 3000 reagent (L3000015, Thermo Fisher Scientific, Tokyo, Japan) following the manufacturer’s instructions. The protein expression of TFAM was lower in tfam-sh1 and tfam-sh2 cells than in control-sh cells, and tfam-sh1 and tfam-sh2 ESCC cells had an approximately 40–60% reduction in mtDNA copy number compared with control-sh cells (Additional file 1: Fig. S1A, B). Additionally, the proliferation rate was significantly lower in tfam-sh1 and tfam-sh2 cells than in control-sh cells (Additional file 1: Fig. S1C).

### Clinical samples

From November 2006 to December 2011, 88 esophageal squamous cell cancer patients underwent R0 resection after neoadjuvant chemotherapy at Osaka University Hospital (Osaka, Japan). Neoadjuvant chemotherapy followed by surgery at our hospital was performed for patients with cStage I (excluding T1N0), II, III, or IV disease without distant organ metastasis. The neoadjuvant chemotherapy regimen consisted of either ACF (adriamycin 35 mg/m^2^, cisplatin 70 mg/m^2^ on Day 1, and continuous fluorouracil 700 mg/m^2^ infusion for 7 days) every 4 weeks or DCF (docetaxel 70 mg/m^2^, cisplatin 70 mg/m^2^ on Day 1, and continuous fluorouracil 700 mg/m^2^ infusion for 5 days) every 3 weeks, as previously described. Surgery was performed after two cycles of neoadjuvant chemotherapy [29]. Clinicopathological findings were classified according to the Union for International Cancer Control (UICC)-TNM classification, seventh edition [30].

Formalin-fixed, paraffin-embedded samples from the patients were collected at our hospital. Cancerous ESCC nests were subjected to DNA extraction after surgery using laser microdissection with a Leica LMD7000 instrument (Leica Microsystems, Wetzlar, Germany). Moreover, of the patients, formalin-fixed, paraffin-embedded samples were collected before and after neoadjuvant therapy from 4 patients with ESCC who had undergone esophagectomy with neoadjuvant therapy. All patients provided written informed consent for the use of the resected specimens. In addition, this study was approved by the ethics committee of Osaka University, Graduate School of Medicine (approval number #15401) and was conducted in accordance with the Declaration of Helsinki.

### Measurement of mtDNA copy number and mRNA expression levels

The mtDNA copy number was measured by quantitative real-time PCR (qPCR) using specific primers for the mtDNA-coded cytochrome oxidase I (MTCO-1) gene and normalized to the expression of the nuclear DNA-encoded cytochrome oxidase IV (COX IV) gene (a mitochondrial respiratory chain enzyme), and the mtDNA copy number was adjusted by setting the mtDNA copy number of control-sh or TE11 cells as 1.00. Complex I/IV-related genes, EMT-related genes including epithelial markers (E-cadherin: CDH1) and mesenchymal markers (N-cadherin: CHD2, vimentin, and Zeb1), and DNA methylation-related genes such as DNMT1, DNMT3A, and DNMT3B were analyzed by qPCR. In addition, the house-keeping gene GAPDH was analyzed in this study. The primers are shown in Additional file 5: Table S1.

### Chemosensitivity assay (viability assay)

A total of 2.0 × 10^3^ cells per well were seeded in 96-well plates, and cell viability was evaluated using Cell Counting Kit-F (CK06, Dojindo, Japan) at 48 h after incubation under chemotherapy, cisplatin (CDDP), 5-fluorouracil (5-FU) and docetaxel (DTX) exposure. The chemotherapy resistance rate was determined as the ratio of the proliferation under chemotherapy at each concentration to that under control treatment. The fluorescence intensity was measured by an iMark™ Microplate Absorbance Reader (BIO-RAD, Tokyo, Japan) using a plate reader at an excitation wavelength of 490 nm and emission wavelength of 515 nm.

### Apoptosis analysis

For the apoptosis assay, an Annexin V-Cy3 Apoptosis Staining/Detection Kit was used (ab14142, Abcam, Cambridge, UK). A total of 2 × 10^5^ cells collected by centrifugation were resuspended in binding buffer, and Annexin V-Cy3 was added. Then, the cells were incubated at room temperature for 5 min in the dark. Flow cytometry was carried out using a FACSCanto II flow cytometer (BD Biosciences). The apoptotic cells were detected as cells bound to Annexin V and propidium Iodide (PI), and early and delay apoptosis cells were collected as respectively low PI and high PI.

### Flow cytometry analysis

Harvested cells (1.0 × 10^5^ cells per well) were seeded in 6-well plates and stained using the MitoProbe JC-1 assay (M34152, Invitrogen) according to the manufacturer’s instructions. The mitochondrial membrane potential (MMP) was analyzed by flow cytometry. The MitoProbe JC-1 assay features a conversion of PE-A (red) to FITC-A (green) when the MMP is depolarized. Flow cytometry was carried out using a FACSCanto II flow cytometer (BD Biosciences), the data were analyzed by FlowJo ver 10.3 software (TOMY DIGITAL BIOLOGY, Tokyo, Japan), and the mean fluorescence intensities (MFI) was measured.

### Fluorescent immunostaining

A total of 2.0 × 10^4^ cells per well were seeded in multiwell glass bottom dishes (D141400, MATSUNAMI, Osaka, Japan). mtDNA in cells was stained with SYBR Green I at a 1:600,000 dilution for 30 min and washed with PBS four times as previously described [31]. Then, the MMP was determined via staining with MitoTracker Orange (M7510, Invitrogen, California, UA) for 10 min followed by two washes with PBS. Fluorescence immunostaining was evaluated by a Confocal Laser Scanning Microscope FV3000 (Olympus, Tokyo, Japan). This fluorescence was quantified by ImageJ v1.53 software, which is an open source and public domain image processing software.

### Complex I and IV activity assays

Complex I activity was investigated using a Complex I Enzyme Activity Microplate Assay Kit (ab109721, Abcam, Cambridge, UK). The protein in cells was added to the microplate wells that had been precoated with a specific capture antibody for complex I. Then, samples were immobilized in the well. In the assay, complex I activity is determined by detecting the oxidation of NADH to NAD + and the simultaneous decrease in dye, which leads to increased absorbance at an optimal density of 450 nm by the SH-9000lab system (Corona Electric, Ibaraki, Japan). Complex IV activity was evaluated using a Complex IV Rodent Enzyme Activity Microplate Assay Kit (ab109911, Abcam, Cambridge, UK). This assay kit was used to determine the activity of cytochrome c oxidase, which participates in complex IV activity, the activity was determined calorimetrically by detecting the oxidation of reduced cytochrome c based on the absorbance change at 550 nm.

### Immunoblotting analysis

Relative protein expression levels were investigated by immunoblotting. E-cadherin, N-cadherin, vimentin, and Zeb-1 expression levels were evaluated using a commercially available EMT antibody sample kit (1:1000 dilution, #9782, Cell Signaling Technology). DNMT1, DNMT3A and DNMT3B levels were evaluated with anti-DNMT1 rabbit monoclonal antibody (1:1000 dilution, #5032, Cell Signaling Technology), anti-DNMT3A rabbit monoclonal antibody (1:1000 dilution, ab227823, Abcam, Cambridge, UK) and anti-DNMT3B rabbit monoclonal antibody (1:1000 dilution, #67259, Cell Signaling Technology), respectively. In addition, TFAM expression levels were assessed using TFAM rabbit polyclonal antibody (1:1000 dilution, ab47517, Abcam, Cambridge, UK). An immunoblotting was quantified by densitometry with ImageJ v1.53 software. The signal intensity for molecule of interest was calibrated by that of β-actin at each point. The relative expression was showed compared to the signal intensity at day0 or control cells as 1.

### Invasion assay

Invasive conditions of cells were measured using 24-well inserted plates with 8 µm membrane pores and Matrigel coating (#354480, Corning, NY, USA). A total of 7.5 × 10^4^ cells (TE11) were added to the upper chamber, and 0.75 ml medium was added in the lower chamber. These cells were incubated at 37 °C in CO2 incubator for 22 h. Thereafter, non-invasive cells in the top chamber were removed by cotton swabs. Invasive cells on the bottom of the membrane were fixed and stained with the Diff-Quick stain kit (16920, Sysmex, Hyougo, Japan). The number of invasive cells was counted.

### DNA methylation assay

The overall DNA methylation in cells was analyzed using a methylated DNA quantification kit (ab117128, Abcam, Cambridge, UK), which quantifies global DNA methylation by specifically measuring the levels of 5-methylcytosine (5-mC) in a microplate-based format. Genomic DNA was applied to microplates and bound to assay wells. Then, capture antibody, detection antibody, enhancer solution and developing solution were added after washing well. The fluorescence intensity was measured with an SH-9000lab instrument (Corona Electric, Ibaraki, Japan) using a plate reader at 450 nm optimal density (OD). The simple calculation of 5-mc percentage in total DNA can be carried out using the following formula: 5-mc% = [(Sample OD − Negative Control OD) ÷ S/(Positive Control OD − Negative Control OD) × 2 ÷ P] × 100%.

### Cell proliferation assay

A total of 5.0 × 10^2^ cells per well were seeded in 96-well plates, and cell viability was evaluated using Cell Counting Kit-F (CK06, Dojindo). The fluorescence intensity was measured at an excitation wavelength of 490 nm and emission wavelength of 515 nm by an iMark™ Microplate Absorbance Reader (BIO-RAD, Tokyo, Japan).

### Immunohistochemistry

Tumor specimens were fixed with 10% formalin, and paraffin-embedded tissue blocks were sectioned into 2.5-μm slices. The sections were deparaffinized with xylene, dehydrated with ethanol in stages and incubated in 10 mM citrate buffer at 110 °C using a pressure cooker for 15 min for antigen removal. Endogenous peroxidase activity of the tissue specimens was blocked by incubating the slides in 3% hydrogen peroxide (H_2_O_2_) dissolved in methanol for 20 min at room temperature. Subsequently, the sample was treated with 1% horse serum albumin for 30 min at room temperature to block nonspecific reactions, all sections were incubated with primary antibodies at 4 °C overnight in a humidified environment. The antibodies used in this study were anti-E-cadherin monoclonal antibody (#3195, dilution 1:100, Cell Signaling Technology), anti-N-cadherin monoclonal antibody (33-3900, dilution 1:100, Invitrogen), and anti-vimentin monoclonal antibody (#5741, dilution 1:300, Cell Signaling Technology). After incubation with secondary antibodies for 20 min at room temperature, the reactions were visualized using the VECTASTAIN^®^ Elite^®^ ABC Kit (PK-6100, Vector Laboratories), which stains the targeted antigen brown, and counterstaining with hematoxylin. The degree of E-cadherin, N-cadherin and vimentin staining was evaluated based on the extent of membranous staining. Five stained hot spots were evaluated (n = 3), and the average of each stained area was estimated by microscopy (BZ-X 710; Keyence).

### TUNEL assay

TUNEL assays were used to investigate apoptosis in formalin-fixed, paraffin-embedded xenograft tumor tissue samples. In brief, paraffin sections (2.5 μm) were deparaffinized in xylene and rehydrated with 70% and 90% alcohol. The TUNEL signal was detected using the ApopTag Fluorescein In Situ Apoptosis Detection Kit (S7110, Sigma–Aldrich). Nuclei were counterstained using VECTASHIELD Mounting Medium with DAPI (Vector Laboratories). Green fluorescence from apoptotic cells was analyzed by fluorescence microscopy (BZ-X 710; Keyence).

### Statistical analysis

The Mann–Whitney U test, the χ^2^ test, or Student’s *t* test was used to compare patient characteristics. The overall survival (OS), including cancer or recurrence death, and recurrence-free survival (RFS) rates from November 2006 to December 2011 were calculated from the date of random assignment, validated by the Kaplan–Meier method, and compared with the log-rank test on an intent-to-treat basis; the corresponding HRs were calculated with the 95% CIs. Cox proportional hazards regression models were used to identify variables significantly associated with prognosis. Continuous variables are expressed as the mean ± SD, unless otherwise stated. Statistical significance was indicated at a* p* value < 0.05. All analyses were performed using JMP^®^ 14 (SAS Institute Inc., Cary, NC, USA).

## Results

### mtDNA copy number in clinical samples of ESCC patients

**Fig. 1:**
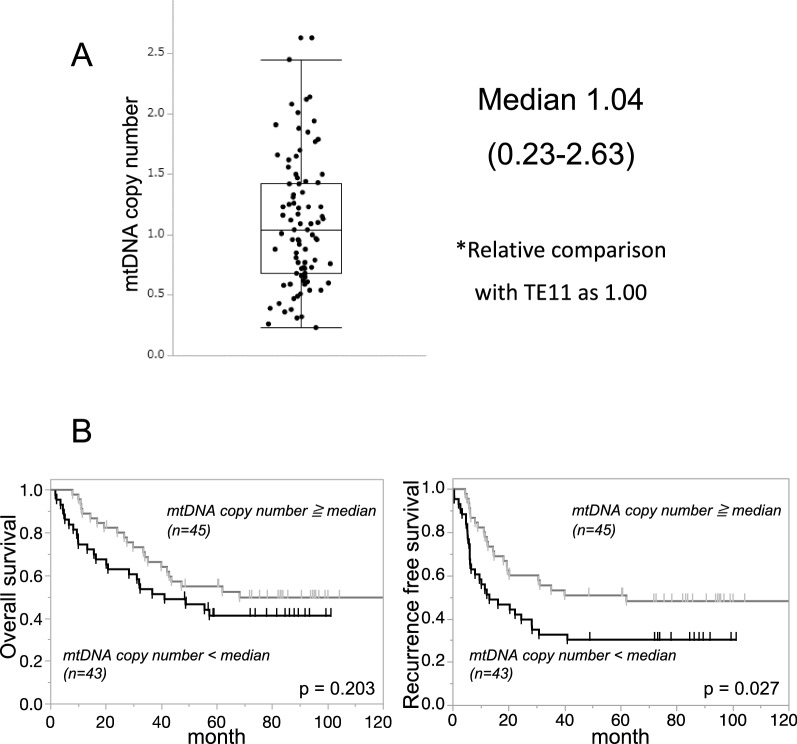
mtDNA copy number in clinical samples from ESCC patients. **A** Patients were divided into two groups: a group with an mtDNA copy number ≥ the median (n = 45) and a group with an mtDNA copy number < the median (n = 43). **B** Overall survival between the mtDNA copy number < median and mtDNA copy number ≥ median groups was not significantly different. However, the mtDNA copy number < median group was significantly associated with shortened recurrence-free survival (p = 0.027)

The patients were divided into two groups: a group with an mtDNA copy number of the median or more (n = 45) and a group with an mtDNA copy number less than the median (n = 43) (Fig. 1A). Table 1 indicates the clinicopathological characteristics. The proportions of samples with cM stage (TNM classification) and pathological response of grade 0 or 1a was significantly higher in the mtDNA copy number < median group than in the mtDNA copy number ≥ median group, and the mtDNA copy number < median group tended have more samples from patients of older age than the mtDNA copy number ≥ median group. Figure 1B shows the Kaplan–Meier estimates of cumulative OS and RFS in the two groups of patients. The mtDNA copy number < the median was significantly associated with shortened RFS (p = 0.027). This study suggests that a low mtDNA copy number is significantly associated with chemotherapy resistance, poor prognosis and metastasis.

**Table 1:** Clinicopathological characteristics

No. of patients		mtDNA copy number ≧ median (n = 45)	mtDNA copy number < median (n = 43)	p value
Age	< 70/70≧	34/11	24/19	0.051
Sex	Male/female	36/9	35/8	0.868
Histology	wel, mod/por	35/10	31/12	0.538
cT	1–2/3, 4	6/39	9/34	0.344
cN	0/1–3	7/38	9/34	0.514
cM	0/1	41/4	32/11	0.049
cStage	I, II/III, IV	11/34	11/32	0.902
NAC regimen	ACF/DCF	34/11	27/16	0.194
Lymphatic invasion	−/+	7/38	5/38	0.592
Venous invasion	−/+	28/17	33/10	0.140
Pathological response	0, 1a/1b, 2	25/20	34/9	0.019
pT	1–2/3, 4	16/29	18/25	0.544
pN	0/1–3	17/28	13/30	0.455
pM	0/1	39/6	37/6	0.933
pStage	I, II/III, IV	21/24	17/26	0.500

*NAC* neoadjuvant chemotherapy, *wel* well differenced type, *mod* moderately differenced type, *por* poor differenced type

### Chemotherapy resistance and mtDNA-depleted ESCC

**Fig. 2:**
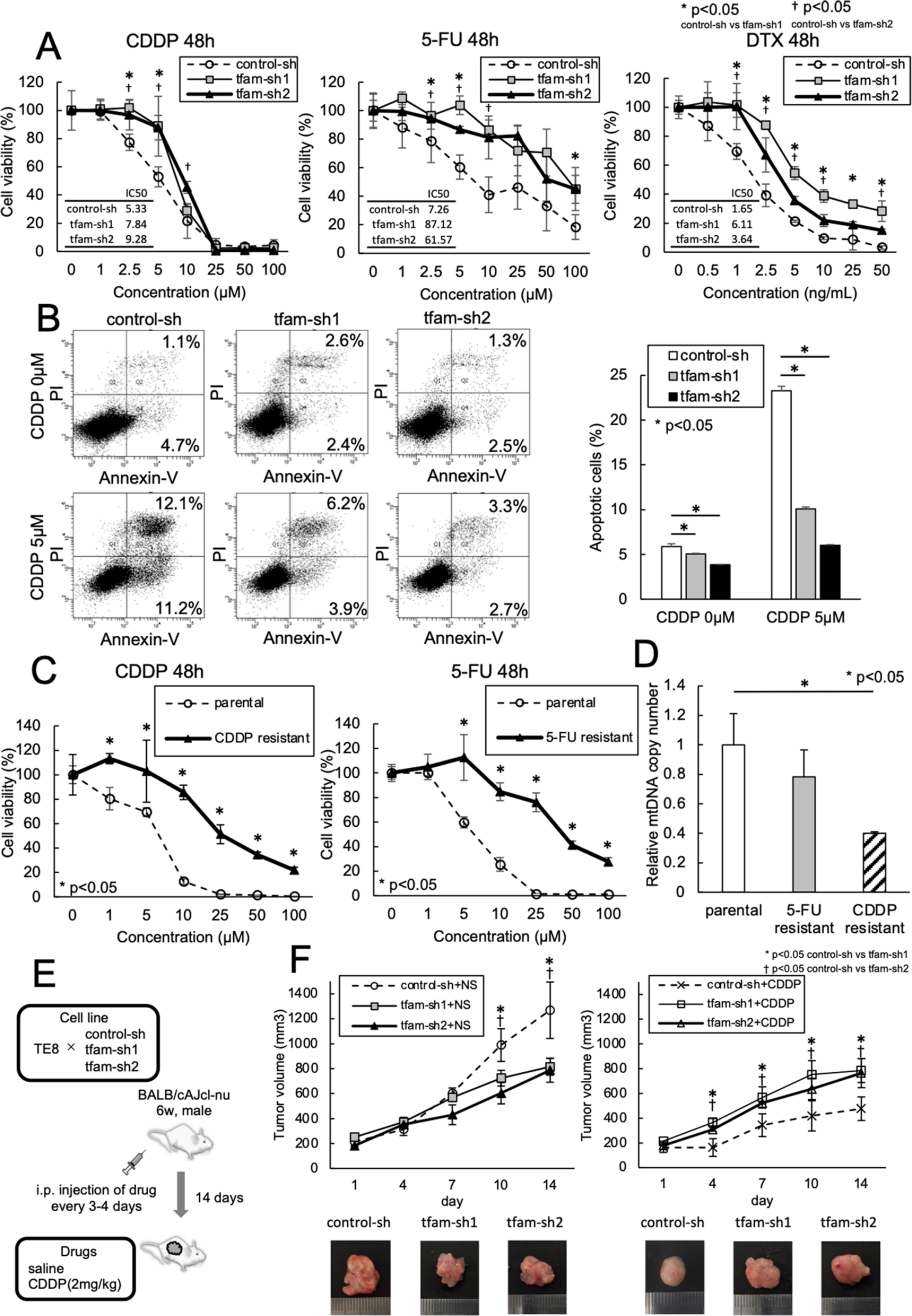
Chemotherapy resistance and mtDNA-depleted ESCC. **A** mtDNA-depleted cells had significantly decreased sensitivity to cisplatin (CDDP), 5-fluorouracil (5-FU), and docetaxel (DTX) compared with control cells. **B** The rate of apoptosis was lower in mtDNA-depleted ESCC than in control cells (apoptosis rate under CDDP 0 µM; control-sh: 5.8%, tfam-sh1: 5.0%, tfam-sh2: 3.8%, apoptosis rate under CDDP 5 µM; control-sh: 23.3%, tfam-sh1: 10.1%, tfam-sh2: 6.0%). **C**, **D** ESCC cells cultured with CDDP and 5-FU long-term were resistant to chemotherapy. Moreover, the mtDNA copy number of CDDP- and 5-FU-resistant cell lines was lower than that of control cells by PCR, and there was significantly difference between CDDP-resistant cell lines and control cells. **E** TE8 control cells and mtDNA-depleted TE8 cells were cultured for 14 days and then injected into BALB/cAJcl nude mice treated with intraperitoneal injection of saline or CDDP every 3–4 days. **F** The proliferation of control cells and mtDNA-depleted ESCC cells was analyzed by WST assay. Control cells in mice treated with saline injection proliferated more than mtDNA-depleted ESCC cells. On the other hand, mtDNA-depleted ESCC cells had higher in tumor volume after CDDP injection than control cells. The physical changes were in line with the results of the WST assay

Figure 2 shows the association between chemotherapy resistance and mtDNA-depleted ESCC. mtDNA-depleted TE11 cells exhibited significantly decreased chemosensitivity to cisplatin (CDDP), 5-fluorouracil (5-FU), and docetaxel (DTX) compared with control TE11 cells (Fig. 2A). The rate of apoptosis was significantly lower in mtDNA-depleted ESCC than in control cells according to an apoptosis assay (apoptosis rate under CDDP 0 µM; control-sh: 5.8%, tfam-sh1: 5.0%, tfam-sh2: 3.8%, apoptosis rate under CDDP 5 µM; control-sh: 23.3%, tfam-sh1: 10.1%, tfam-sh2: 6.0%) (Fig. 2B). These results suggested that mtDNA-depleted ESCC cells were resistant to chemotherapy. The cell lines were cultured for a long time under CDDP and 5-FU exposure, and these cell lines were resistant to chemotherapy (Fig. 2C). Moreover, the mtDNA copy number of CDDP- and 5-FU-resistant cell lines was significantly lower than that of control cells (Fig. 2D). In the in vivo experiment, the proliferation of control cells injected in saline was higher than that of mtDNA-depleted ESCC cells. On the other hand, mtDNA-depleted ESCC cells had higher in tumor volume after CDDP injection than control cells (Fig. 2E, F). The above results suggest that ESCC cells with chemotherapy resistance have decreased mtDNA copy number and that depletion of mtDNA induces chemotherapy resistance.

### Association between mtDNA and mitochondrial membrane potential

**Fig. 3:**
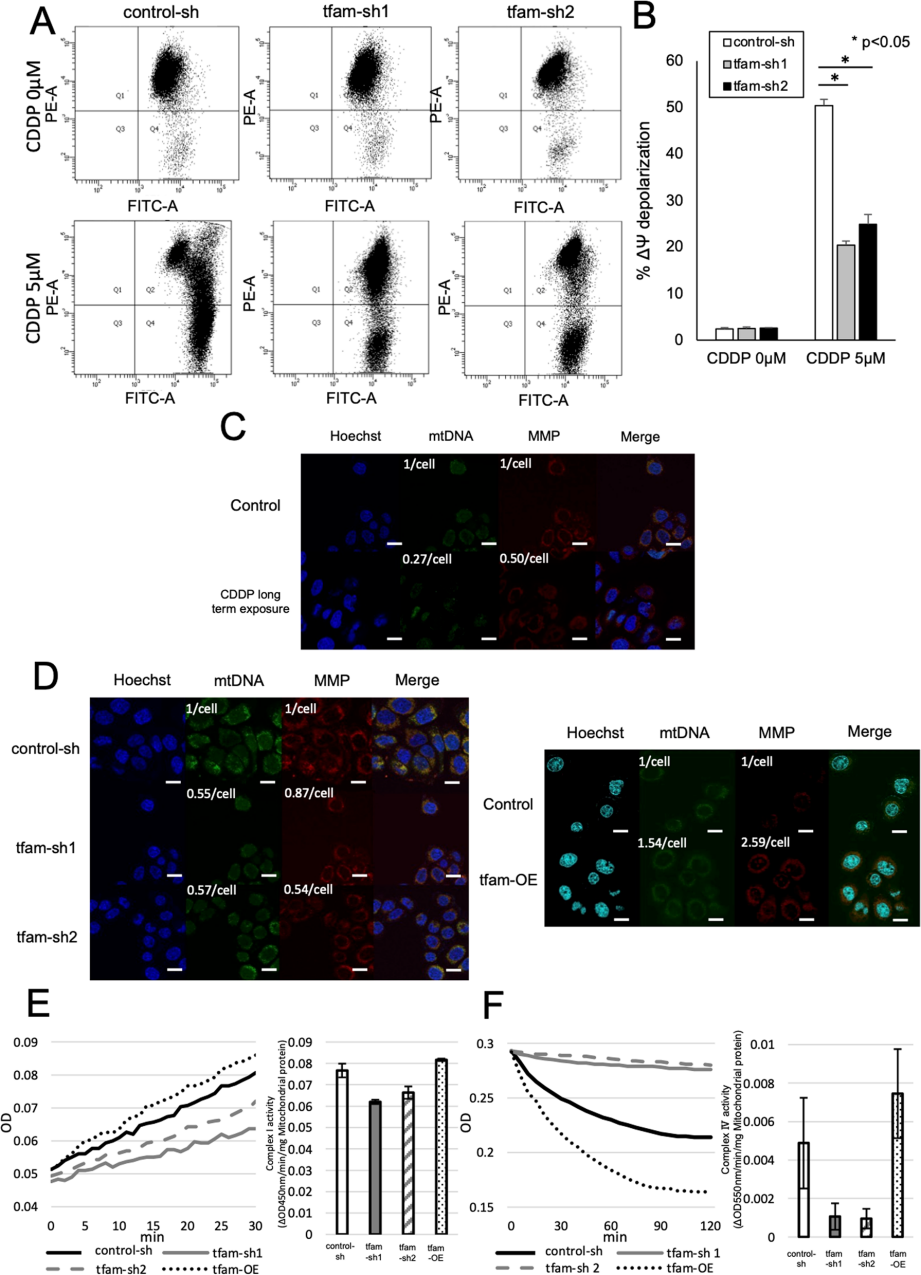
Association between mtDNA and mitochondrial membrane potential. **A** The rate of transition from PE-A to FITC-A after 5 µM CDDP administration was lower in mtDNA-depleted ESCC cells than control cells, and the depolarization of mitochondrial membrane potential was significantly lower in mtDNA-depleted ESCC cells than control cells. **B** The ESCC cell line which was exposure CDDP for long term had lower the fluorescence of mtDNA and mitochondrial membrane potential (MMP) than control cell lines. Scale bar, 20 µM. Quantification of mtDNA and MMP fluorescence per cell (quantification/cell). **C** ESCC cells which was administrated CDDP for long time had lower fluorescence of mtDNA and MMP compared control cells. **D** There was lower fluorescence of mtDNA and MMP in mtDNA-depleted ESCC cells than control cells. On the other hand, mtDNA-increased ESCC cells had higher fluorescence of mtDNA and MMP than control cells. Scale bar, 20 µM. Quantification of mtDNA and MMP fluorescence per cell (quantification/cell). **E**, **F** The complex activity assay kit showed that mtDNA-depleted ESCC cells reduced complex I and IV activity assay compared to control cells. On the other hand, there was higher complex I and IV activity in mtDNA-increased ESCC cells than control cells

MMP is an important indicator of mitochondrial function [32, 33]. Hence, we focused on the associations between MMP, mtDNA copy number and EMT. CDDP induced a transition of PE-A to FITC-A in control cells and mtDNA-depleted ESCC cells. The rate of transition from PE-A to FITC-A after CDDP administration was lower in mtDNA-depleted ESCC cells than in control cells (Fig. 3A), and the MMP depolarization rate after CDDP administration was significantly lower in mtDNA-depleted ESCC cells than in control cells (Fig. 3B). ESCC cells administered CDDP for a long time had lower fluorescence, indicating lower mtDNA and MMP, than control cell lines (Fig. 3C). Therefore, the data suggest that chemotherapy induces a decrease in MMP. Additionally, there was lower fluorescence, and thus lower mtDNA and MMP, in mtDNA-depleted ESCC cells than in control cells. On the other hand, ESCC cells with increased mtDNA had higher fluorescence, indicating higher mtDNA and MMP, than control cells (Fig. 3D). The quantification of mtDNA and MMP fluorescence per cell was in line with the above results. Complexes I and IV, which are electron-transferring enzymes, are the main factors regulating the MMP [34]. Figure 3E and F show that mtDNA-depleted ESCC cells had reduced complex I and IV activity compared to control cells; this decreased activity regulated the MMP, and there was higher complex I and IV activity in ESCC cells with increased mtDNA than in control cells. These results indicated that mtDNA controls the MMP via complexes I and IV. Hence, the decreased mtDNA induced by chemotherapy leads to a low MMP. Moreover, the mRNA expression levels of complexes I and IV were correlated with mtDNA copy number (Additional file 2: Fig. S2A).

### Mitochondrial membrane potential and the epithelial–mesenchymal transition

**Fig. 4:**
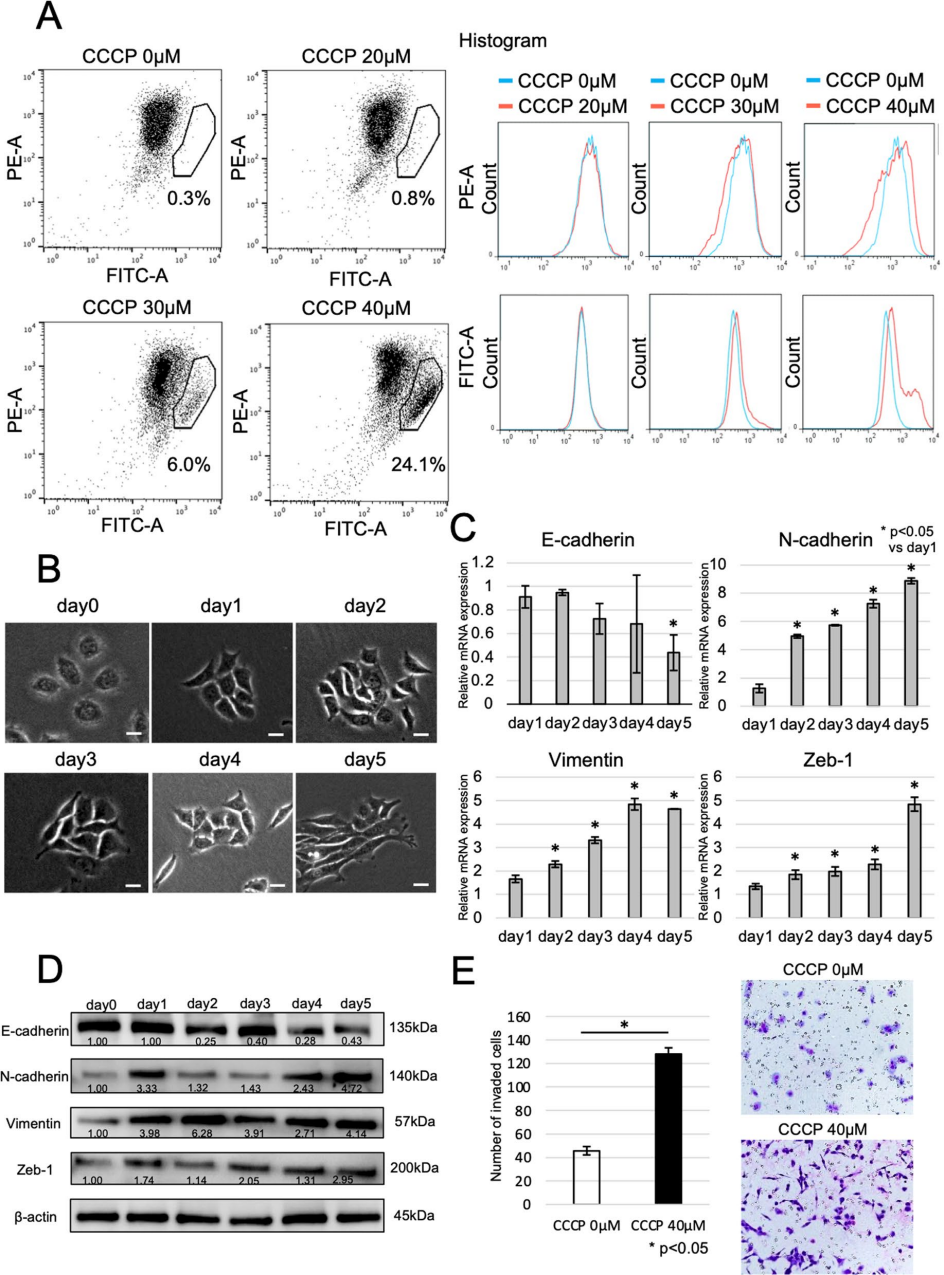
Mitochondrial membrane potential and the epithelial–mesenchymal transition. **A** The association between carbonyl cyanide m-chlorophenylhydrazone (CCCP), which artificially reduces mitochondrial membrane potential, and ESCC cells was investigated by FACS. Of the concentrations of CCCP administered (20 µM, 30 µM and 40 µM), 40 µM CCCP induced the greatest decrease in mitochondrial membrane potential. **B** TE11 cells exposed to 40 µM CCCP showed spindle cell transformation, and the severity of transformation depended on the exposure time (Days 1–5). Scale bars, 20 µM. **C** Compared with control cells, ESCC cells cultured with CCCP showed significantly decreased relative mRNA expression of E-cadherin at Day 5, while the relative mRNA expression level of N-cadherin, vimentin, and Zeb-1 in ESCC cells was significantly increased. **D** Compared with that in control cells (Day 0), the protein expression level of E-cadherin at Day 5 after CCCP exposure was decreased. On the other hand, the protein expression levels of N-cadherin, vimentin, and Zeb-1 at day 5 after CCCP exposure were increased. *The densitometry of relative protein expression. **E** The number of invasive cells were significantly higher in TE11 which administrated 40 µM CCCP for 5 days than in TE11 without CCCP (CCCP 0 µM)

The uncoupler carbonyl cyanide m-chlorophenylhydrazone (CCCP; ab141229, Abcam, Cambridge, UK), which is a compound that inhibits the conjugation of both reactions in oxidative phosphorylation, artificially reduces mitochondrial membrane potential. This study investigated the influence of CCCP on ESCC cells by FACS. Exposure to 40 µM CCCP for 5 days induced the greatest decrease (24.1%) in mitochondrial membrane potential at 20–40 µM (Fig. 4A). The mtDNA copy number in cells exposed to 40 µM CCCP was not different from that in control cells not exposed to CCCP (Additional file 2: Fig. S2B). Cells that were exposed to 40 µM CCCP showed spindle cell transformation, which varied in severity depending on the exposure time (Days 1–5) (Fig. 4B). The relative mRNA expression levels of E-cadherin in ESCC cells cultured with CCCP were significantly decreased at Day 5 compared with those in control cells not treated with CCCP. On the other hand, ESCC cells treated with CCCP had significantly higher mRNA expression of N-cadherin, vimentin, and Zeb-1 than those not treated with CCCP (Fig. 4C). Additionally, the protein expression level of E-cadherin at Day 5 after CCCP exposure was decreased compared with that in control cells (Day 0), and the N-cadherin, vimentin, and Zeb-1 protein expression levels at Day 5 after CCCP exposure were increased compared with those in control cells (Day 0) (Fig. 4D). These results regarding the relationship between MMP and EMT were similar in TE8 cells (Additional file 2: Fig. S2D, E), and treatment with 10 µM CCCP for 5 days reduced mitochondrial membrane potential (Additional file 2: Fig. S2C). Moreover, invasion assay showed that TE11 which administrated CCCP for 5 days had significantly more invasion than TE11 without CCCP (Fig. 4E). These results suggest that decreased MMP induces EMT.

### Relationship of mtDNA and mitochondrial membrane potential with DNA methylation

**Fig. 5:**
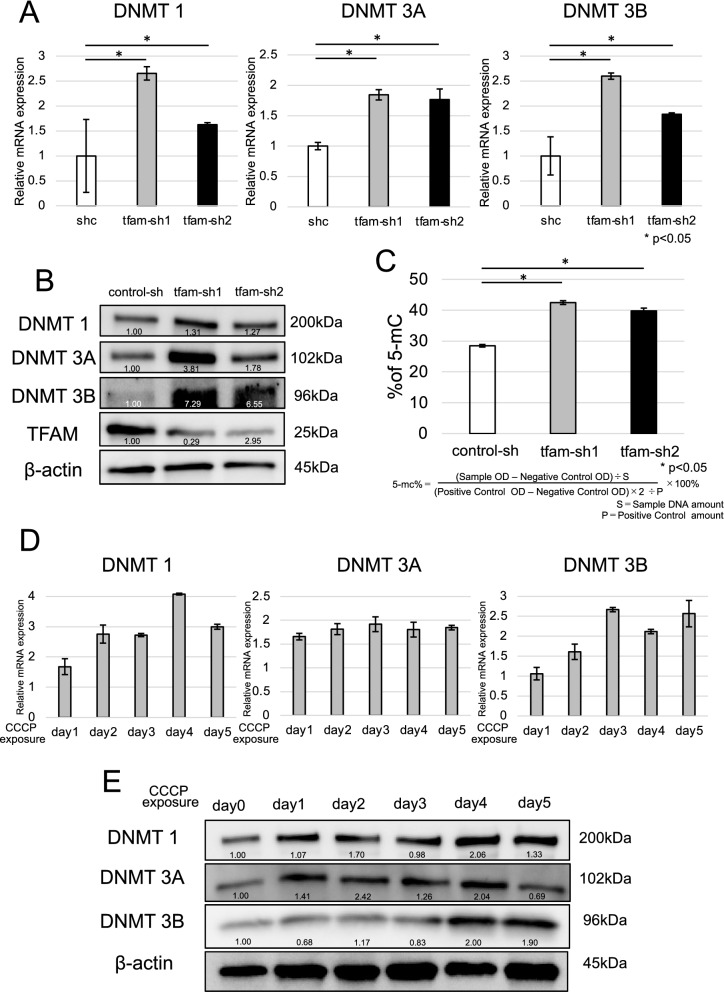
Relationship between mtDNA, mitochondrial membrane potential and DNA methylation. **A** mtDNA-depleted TE11 cells had approximately twofold higher mRNA expression levels of DNMT1, DNMT3A and DNMT3B than control cell lines. **B** mtDNA-depleted TE11 cells had higher protein expression levels of DNMT1, DNMT3A and DNMT3B than control cells. Control-sh cells had higher protein expression of TFAM that tfam-sh cells. *The densitometry of relative protein expression. **C** mtDNA-depleted TE11 cells had approximately 10% more overall DNA methylation than control cell lines. **D** The mRNA expression levels of DNMT1, DNMT3A and DNMT3B were approximately 100–200% higher in CCCP-exposed cell lines than in control cell lines. **E** There were higher protein expression levels of DNMT1, DNMT3A and DNMT3B in TE11 cells exposed to 40 µM CCCP than in control cells. *The densitometry of relative protein expression

This study additionally focused on DNA methylation and the methylation transcription factor DNA methyltransferase (DNMT) in detail mechanism which depleted mtDNA and decreased MMP controlled the expression of nuclear genes. mtDNA-depleted TE11 cells had an approximately twofold higher mRNA level of DNMT1, DNMT3A and DNMT3B than the control cell lines (Fig. 5A). The DNMT1, DNMT3A and DNMT3B protein levels were higher in ESCC cells with low mtDNA than in control cells (Fig. 5B). mtDNA-depleted TE11 cells hain DNMT1, DNMT3a and DNMT 3Bd an approximately 10% higher 5-mC level (a measure of global DNA methylation) than control cell lines (Fig. 5C). Hence, low mtDNA copy number induced DNA methylation by DNMT. The mRNA expression level of DNMT1, DNMT3A and DNMT3B was twofold to threefold higher in ESCC cell lines treated with CCCP than in those not treated with CCCP (Fig. 5D). There were almost higher protein levels of DNMT1, DNMT3A and DNMT3B in ESCC cell lines exposed to 40 µM CCCP than in ESCC cell lines that were not exposed to 40 µM CCCP (Fig. 5E). mtDNA-depleted TE8 cells had higher mRNA and protein levels of DNA methylation-related genes, such as DNMT1, DNMT3A and DNMT3B (Additional file 3: Fig. S3A, B). These data indicate that decreased mtDNA and MMP promote DNMT expression via DNA methylation.

### The influence of DNMT inhibitors on chemotherapy resistance

**Fig. 6:**
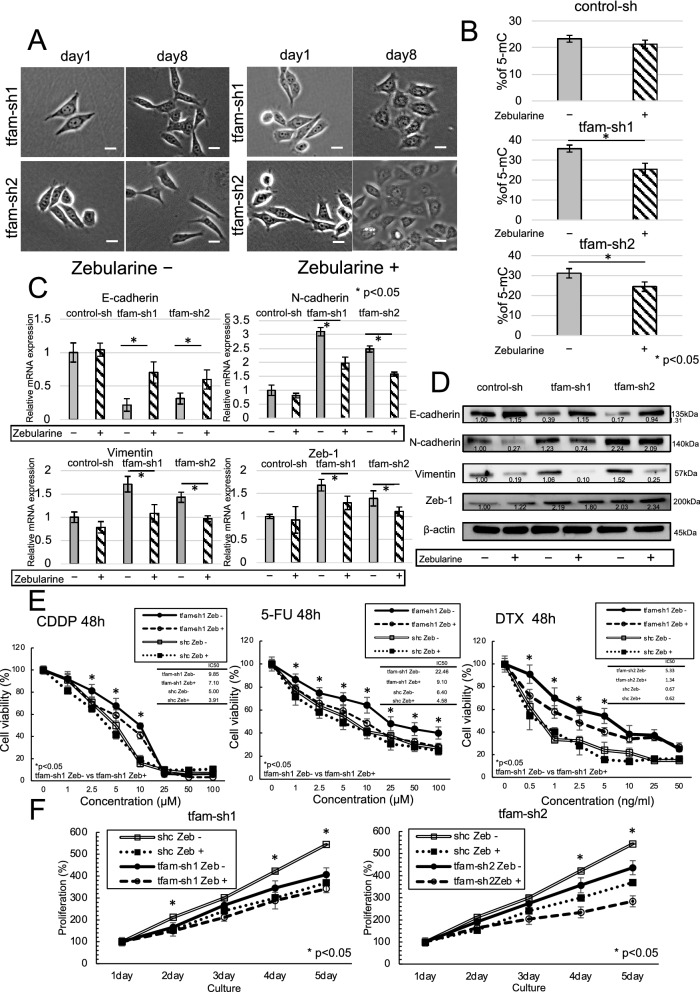
mtDNA-depleted ESCC and a DNMT inhibitor. **A** mtDNA-depleted ESCC cells treated with zebularine showed transformation from spindle to nonspindle cells. **B** The overall DNA methylation level was 10% lower after zebularine treatment in mtDNA-depleted ESCC cells. **C** There was similar expression of E-cadherin, N-cadherin, vimentin, and Zeb-1 in control cell lines. On the other hand, the expression of E-cadherin was higher after administration of zebularine in mtDNA-depleted ESCC cells, and mtDNA-depleted ESCC cells treated with zebularine had lower expression of N-cadherin, vimentin and Zeb-1 than cells not treated with zebularine. **D** The protein level of E-cadherin was increased by zebularine treatment in mtDNA-depleted ESCC cells, and the protein levels of N-cadherin and vimentin were decreased by zebularine treatment. *The densitometry of relative protein expression. **E** Zebularine administration to mtDNA-depleted ESCC cells significantly increased sensitivity to CDDP, 5-FU and DTX, while there was no difference in chemosensitivity in control cell lines treated with and not treated with zebularine. **F** The proliferation rates of mtDNA-depleted cells and control cells were significantly reduced by zebularine

ESCC cells depleted of mtDNA showed cell transformation from spindle to nonspindle morphology after administration of 100 µM zebularine (S7113, Selleck, Houston, USA), a DNMT inhibitor, for 8 days (Fig. 6A). Also, the morphology of control cell with DNMT inhibitor remained nonspindle and had no change (Additional file 3: Fig. S3C). 5-mC was decreased by 10% in mtDNA-depleted ESCC cells treated with zebularine compared to that in ESCC cells not treated with zebularine (Fig. 6B). The mRNA expression of E-cadherin, N-cadherin, vimentin, and Zeb-1 was similar in control cell lines treated with and not treated with zebularine. However, the mRNA expression of E-cadherin was significantly higher in mtDNA-depleted ESCC cells treated with zebularine than in those not treated with zebularine, and mtDNA-depleted ESCC cells treated with zebularine had significantly lower expression of N-cadherin and vimentin than cells not treated with zebularine (Fig. 6C). Additionally, the protein expression of E-cadherin was increased by zebularine, and the protein levels of N-cadherin and vimentin were decreased by zebularine (Fig. 6D). Zebularine significantly impacted mtDNA-depleted ESCC cells by increasing chemosensitivity to CDDP, 5-FU and DTX, while there was no difference in chemosensitivity in control cell lines treated with and not treated with zebularine (Fig. 6E). The proliferation rates of mtDNA-depleted ESCC cells and control cells were significantly reduced by zebularine (Fig. 6F). Based on these results, the DNMT inhibitor zebularine improves chemotherapy sensitivity by suppressing EMT.

**Fig. 7:**
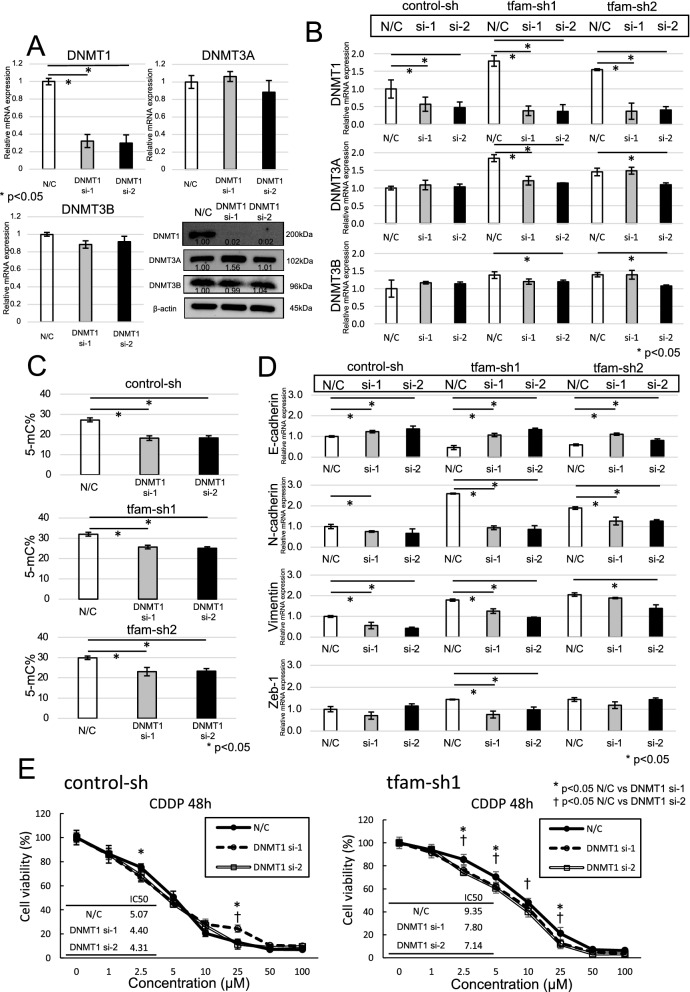
The impact of DNMT1 expression on mtDNA-depleted ESCC cells. **A** Depletion of mtDNA in TE11 cells by DNMT1 knockdown. *The densitometry of relative protein expression. **B** The mRNA expression levels of DNMT1 in control cells and mtDNA-depleted cells were significantly lower after DNMT1 knockdown, and the DNMT3A and DNMT3B mRNA expression levels were not different in DNMT1 knockdown cells. **C** The overall DNA methylation level was reduced by approximately 7–8% by DNM1 knockdown. **D** DNMT1 knockdown in mtDNA-depleted ESCC cells significantly increased E-cadherin mRNA expression compared to that in cells without DNMT1 knockdown. In addition, the mRNA expression levels of N-cadherin and vimentin were significantly lower in DNMT1 knockdown cells than in control cells. **E** mtDNA-depleted ESCC cells with DNMT1 knockdown had increased sensitivity to CDDP compared with cells without DNMT1 knockdown

Moreover, this study evaluated the impact of DNMT1 expression on mtDNA-depleted ESCC cells. DNMT1 knockdown (DNMT1-siRNA, AM16708, Thermo Fisher Scientific) reduced the mRNA and protein expression of DNMT1 in ESCC cells, although the expression of DNMT3A and DNMT3B was similar after DNMT1 knockdown (Fig. 7A). The mRNA expression levels of DNMT1 in control cells and mtDNA-depleted cells were significantly lower after DNMT1 knockdown. However, the mRNA expression levels of DNMT3A and DNMT3B after DNMT1 knockdown were low reduction rate compared that of DNMT1 (Fig. 7B). Overall DNA methylation was reduced by approximately 7–8% by DNMT1 knockdown (Fig. 7C). mtDNA-depleted ESCC cells with DNMT1 knockdown had significantly increased mRNA expression levels of E-cadherin compared to cells without DNMT1 knockdown. In addition, N-cadherin and vimentin mRNA expression was significantly lower in DNMT1 knockdown cells than in cells without DNMT1 knockdown (Fig. 7D). The protein levels change of DNMT and EMT markers were similar to mRNA level expression in DNMT1 knockdown (Additional file 3: Fig. S3D). The mtDNA-depleted ESCC cells with DNMT1 knockdown had significantly increased sensitivity to CDDP compared with cells without DNMT1 knockdown (Fig. 7E). Therefore, we suggest that DNMT1 knockdown suppresses chemotherapy resistance by increasing epithelial markers and decreasing mesenchymal markers.

### Use of a DNMT inhibitor to overcome chemotherapy resistance in a preclinical trial

**Fig. 8:**
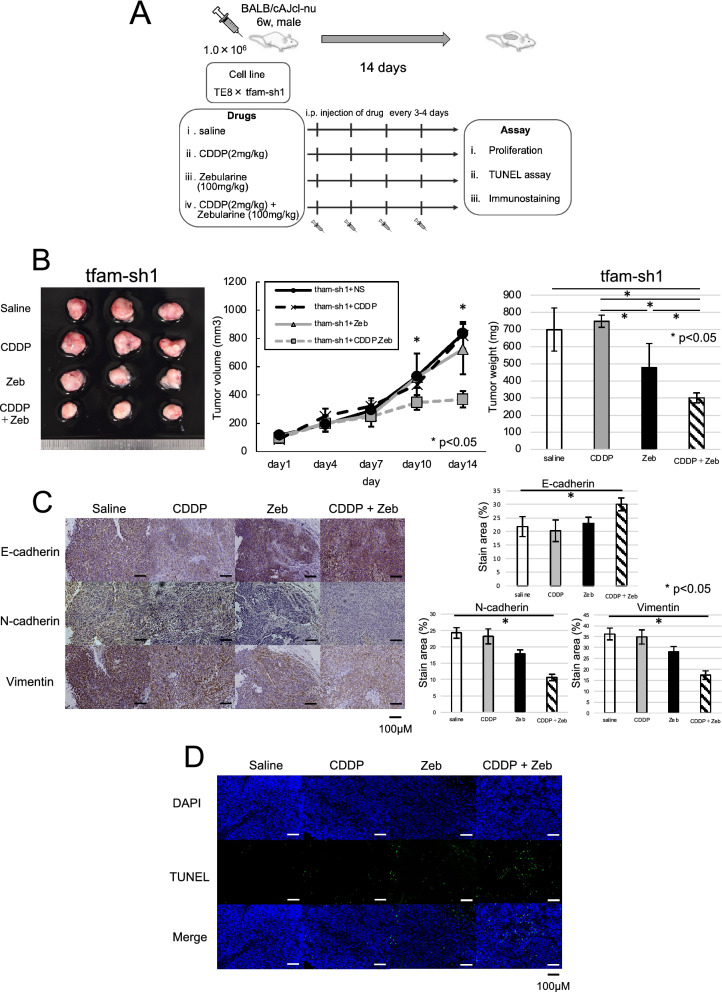
A DNMT inhibitor improves chemotherapy sensitivity in a preclinical trial. **A** Control cells and mtDNA-depleted ESCC TE11 cells were allowed to grow for 14 days in BALB/cAJcl nude mice, after which saline, CDDP, zebularine or CDDP combined with zebularine were injected intraperitoneally every 3–4 days. **B** Regarding physical changes, there was no difference in tumor volume between the saline- and CDDP-treated mice that received mtDNA-depleted ESCC cells. However, the tumor volume was lower in mice that received cells followed by injection of CDDP combined with zebularine than in mice that received cells followed by injection of only CDDP or zebularine. The proliferation of mtDNA-depleted ESCC cells in mice treated with CDDP combined with zebularine was significantly lower than that of cells in mice treated with only CDDP or zebularine. The tumor weights were in line with the above results. **C** Immunostaining showed that the expression of E-cadherin was higher in mtDNA-depleted ESCC cells in mice treated with CDDP and zebularine than in those in mice treated with other drugs and that the expression of N-cadherin and vimentin was lower in those in mice treated with CDDP and zebularine than in those in mice treated with other drugs. **D** CDDP combined with zebularine induced the highest apoptosis rate of the drugs

The mtDNA copy number of the ESCC patients after NAC was approximately 40% lower than that of the ESCC patients before NAC (Additional file 4: Fig. S4A). Therefore, we hypothesized that a DNMT inhibitor could improve chemotherapy sensitivity in mtDNA-depleted ESCC cells with chemotherapy resistance. mtDNA-depleted ESCC cells were injected subcutaneously into BALB/cAJcl nude mice (n = 3), and subcutaneous tumors were administered the following drugs 7 days after injection: saline, CDDP, zebularine and CDDP combined with zebularine intraperitoneally every 3–4 days (Fig. 8A). The physical change in tumor volume in was not different between the saline- and CDDP-treated mice that received mtDNA-depleted ESCC cells. However, the tumor volume was lower in mice that received cells and were then treated with CDDP combined with zebularine than in mice that received cells and were then treated with only CDDP or zebularine. Moreover, the proliferation of mtDNA-depleted ESCC cells in mice treated with CDDP combined with zebularine was significantly lower than that of cells in mice treated with only CDDP or zebularine. The tumor weight results were in line with the above results (Fig. 8B). Immunostaining showed that E-cadherin expression was higher in mtDNA-depleted ESCC cells injected into mice treated with CDDP combined with zebularine than in those injected into mice treated with other drugs and that the expression of N-cadherin and vimentin was lower in cells injected into mice treated with CDDP combined with zebularine than in cells injected into mice treated with other drugs (Fig. 8C). Compared with other drugs, CDDP combined with zebularine induced the most apoptosis (Fig. 8D). Our results show that chemotherapy combined with a DNMT inhibitor reduces EMT and improves chemotherapy sensitivity in ESCC cells with low mtDNA and chemotherapy resistance.

## Discussion

**Fig. 9:**
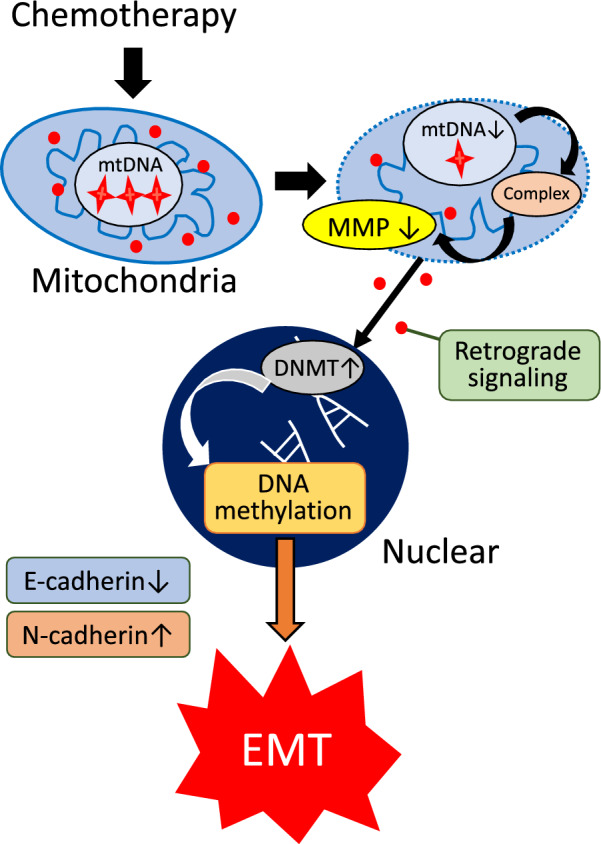
Mitochondrial membrane potential and DNA methylation underly the relationship between mtDNA and epithelial–mesenchymal transition. Depletion of mtDNA copy number reduces the mitochondrial membrane potential via mitochondrial complexes, and a decrease in the membrane potential induces DNA methylation by increasing DNMT. As a result, a low mtDNA copy number has an impact on epithelial–mesenchymal transition

This study found that MMP change was an important factor in the mechanism by which mtDNA depletion affects chemotherapy resistance, and DNA methylation induced by DNMT expression was associated with EMT. Several studies have reported that mitochondrial dysfunction induced by mtDNA depletion is related to the activation of EMT, which induces therapeutic resistance via mitochondrial retrograde signaling in cancer cells [26, 35, 36]. Retrograde signaling is a transduction pathway running from mitochondria to the nucleus under normal and pathophysiological conditions [37]. Based on our results, retrograde signaling, which induces DNA methylation in the nucleus and EMT, originates in the inner mitochondrion and travels to the cytoplasm by reducing MMP. We showed the detailed mechanism underlying the association between mtDNA depletion and EMT (Fig. 9). Moreover, a DNMT inhibitor improved chemotherapy resistance by decreasing mtDNA copy number in ESCC cells and has the potential to be a new treatment agent.

We showed the correlation between decreased mtDNA copy number and poor response to chemotherapy both in clinical samples and in vitro. A previous study reported that depletion of mtDNA contributes to chemoresistance development in several malignant tumors [38, 39], and our study is consistent with this finding. On the other hand, a reduced mtDNA copy number increases the chemotherapy sensitivity of tumor cells in cancers such as nasopharyngeal cancer [40]. Hence, the association between mtDNA copy number and chemotherapy resistance in cancer is controversial.

The mechanism by which a decreased mtDNA copy number induces treatment resistance has been shown to involve chemically induced mtDNA depletion, which suggests the potential existence of an apoptosis-resistant phenotype in advanced cancer [41–43], and a depleted mtDNA copy number suppresses ROS production to decrease the sensitivity to cisplatin in ovarian cancer [39]. Conversely, a previous study reported that a low mtDNA copy number increased ROS levels in tumor cells and the apoptosis rate induced by chemotherapy. Additionally, a low mtDNA copy number has been shown to affect mitochondrial dysfunction in several cancers [44], and colon cancer cells with a low mtDNA copy number show a decreased oxygen consumption rate and respiratory control ratio, which are markers of mitochondrial function [45].

MMP, an important indicator of mitochondrial function, is generated by the potential difference due to the concentration gradient of H^+^ across the inner membrane and has an essential role in energy storage during oxidative phosphorylation [33]. A previous study showed that chemotherapy, including 5-FU and vincristine, decreased MMP in hepatocellular carcinoma and lymphoid leukemia [46, 47]. Additionally, MMP depolarization was found to be related to cancer cell resistance to treatments such as chemotherapy and radiotherapy [48, 49]. We found that a decrease in the mitochondrial membrane potential induced EMT, which was associated with treatment resistance in ESCC. Additionally, MMP is controlled by proton pumps, including mitochondrial complexes, which are electron-transferring enzymes [34]. We showed that decreased mtDNA copy number reduced the expression and activity of complexes I and IV, which are encoded by mtDNA. Therefore, the results of our study are consistent. No study has shown an association between MMP and EMT in cancer. The present study is the first to indicate the detailed mechanism underlying the relationships between MMP, mtDNA and EMT. However, it is necessary to investigate the influence of retrograde signaling on EMT in ESCC.

DNA methylation is associated with histone modifications, and the interplay of these epigenetic modifications is crucial for regulating genome function via alterations in chromatin structure [50, 51]. The covalent addition of methyl groups usually occurs at cytosines within CpG dinucleotides, which are concentrated in large clusters called CpG islands. DNMTs are responsible for establishing and maintaining the methylation pattern. No previous study has reported an association between MMP changes and DNMT expression, and thus, this study has very novel findings. However, we were not able to evaluate the detailed mechanism by which MMP changes induce DNMT expression. Several studies have shown that the mtDNA copy number influences specific nuclear DNA methylation, resulting in differential expression of specific genes, and these findings might support the results of our study [52, 53]. On the other hand, the relationship between DNMT expression and EMT has been reported: DNA methylation induced EMT and was associated with resistance to sorafenib in patients with advanced hepatocellular carcinoma [54]. Moreover, Xie C et al. reported that depletion of mtDNA led to a hypermethylated E-cadherin promoter via DNA methylation by DNMT1 in prostate cancer and that hypermethylation reduced E-cadherin expression [55], and Cui et al. suggested that DNMT3A contributes to suppressing E-cadherin expression via DNA hypermethylation and the H3K9me2 and H3K27me3 histone modifications in gastric cancer [56]. Based on these previous findings, the mechanism underlying the association between DNMT and EMT identified in our study might be associated with promoter hypermethylation and histone modification.

Moreover, in the present study, a DNMT inhibitor combined with chemotherapy reduced the induction of EMT and improved chemotherapy sensitivity of ESCC cells with a low mtDNA copy number, which otherwise had chemotherapy resistance and high metastasis activity in vitro and in vivo. The advanced ESCC with chemoresistance progresses tumor volume during chemotherapy and has poor prognosis. We previously showed that ESCC cells with a low mtDNA copy number had strong cancer stemness and mesenchymal characteristics [13]. In addition, in this study, these cells were resistant to chemotherapy. The present study suggests that DNMT inhibitors are able to inhibit EMT and improve chemotherapy sensitivity, and the combination of DNMT inhibitors with chemotherapy may reduce tumor volume and improve prognosis for advanced ESCC with chemotherapy resistance. Therefore, the combination of DNMT inhibitors with chemotherapy and the development of drugs that restore mtDNA copy number could provide selective therapy targeting cancer stem cells and cancer cells with chemotherapy resistance. These treatments have the potential to represent a paradigm shift in therapeutic strategies for cancers.

## Conclusion

The present study showed that the mtDNA copy number affects EMT via changes in the MMP and DNA methylation in ESCC. Therapeutic strategies increasing the mtDNA copy number and suppressing DNMT may be effective for preventing EMT and treatment resistance.

## Supplementary Information

**Additional file 1: Figure S1.** The association between TFAM knockdown and mtDNA copy number. **A** TFAM protein expression was lower in tfam-sh than control-sh TE8 cells. tfam-sh1 and tfam-sh2 TE8 cells had an approximately 60% decrease in mtDNA copy number compared with control-sh TE8 cells. B: tfam-sh cells had lower protein expression levels of TFAM than control-sh cells. The mtDNA copy number in tfam-sh1 and tfam-sh2 TE11 cells was approximately 40% lower than that in control-sh TE11 cells. C: The proliferation rates of tfam-sh1 and tfam-sh2 cells were significantly lower than that of control-sh cells.

**Additional file 2: Figure S2.** Mitochondrial membrane potential and epithelial–mesenchymal transition. **A** The complex I and IV mRNA expression levels was lower in mtDNA-depleted ESCC and was higher in mtDNA-increased ESCC. **B** The ESCC cells had no difference in mtDNA copy number under 40 μM CCCP exposure. **C** 30 µM CCCP induced to most decrease in mitochondrial membrane potential. **D** TE8 under 30 µM CCCP exposure showed spindle cell transformation, depending on exposure time (day1-5). Scale bars, 20 µM. **E** The relative mRNA expression level of E-cadherin in ESCC cells which cultured with CCCP was significantly decreased at day5 compared with control cells in TE8. The relative mRNA expression level of N-cadherin, vimentin, and zeb-1 in its cells was significantly increased compared with control cells.

**Additional file 3: Figure S3.** Relationship of mtDNA and DNA methylation. **A** The mtDNA-depleted of TE8 cells had appropriately double higher the mRNA expression level of DNMT-1, DNMT-3A and DNMT-3B than control cell lines. **B** A mtDNA-depleted of TE8 cells had higher protein expression levels of DNMT-1 and DNMT-3A compared with control cells. **C** The control cell morphology with DNMT inhibitor remained nonspindle and unchanged. Scale bars, 20 µM. **D** The DNMT1 protein level in control cells and mtDNA-depleted cells were lower expression by DNMT1 knockdown, and DNMT3A and 3B protein levels had little change in DNMT1 knockdown cell. Also, the protein level of E-cadherin was higher in mtDNA-depleted cells of DNMT1 knockdown. The N-cadherin and vimentin protein levels by DNMT1 knockdown had lower expression in mtDNA-depleted ESCC cells.

**Additional file 4: Figure S4.** The change in mtDNA copy number after NAC. **A** The mtDNA copy number of the ESCC patients after NAC was about 40% lower than this patients before NAC.

**Additional file 5: Table S1.** Primer used in this study.

## Data Availability

The data supporting the conclusions of this article are presented within the article and its additional files.

## Abbreviations

ESCC: Esophageal squamous cell carcinoma
ATP: Adenosine triphosphate
OXPHOS: Oxidative phosphorylation
ROS: Reactive oxygen species
mtDNA: Mitochondrial DNA
EMT: Epithelial-mesenchymal transition
TFAM: Mitochondrial transcription factor A
qPCR: Quantitative real-time PCR
MTCO-1: MtDNA-coded cytochrome oxidase I
COX IV: Cytochrome oxidase IV
CDH1: E-cadherin
CDH2: N-cadherin
CDDP: Cisplatin
5-FU: 5-Fluorouracil
DTX: Docetaxel
MFIs: Mean fluorescence intensities
PI: Propidium Iodide
MMP: Mitochondrial membrane potential
5-mC: 5-Methylcytosine
H2O2: Hydrogen peroxide
CCCP: Carbonyl cyanide m-chlorophenylhydrazone
DNMT: DNA methyltransferase
