# Supplementary figures and images for: Low mitochondrial DNA copy number induces chemotherapy resistance via epithelial-mesenchymal transition by DNA methylation in esophageal squamous cancer cells
Source: J Transl Med. 2022 Aug 29;20:383. doi: 10.1186/s12967-022-03594-2 (PMC9422107; doi:10.1186/s12967-022-03594-2)

Supplemental Figure. 1

A

TE8

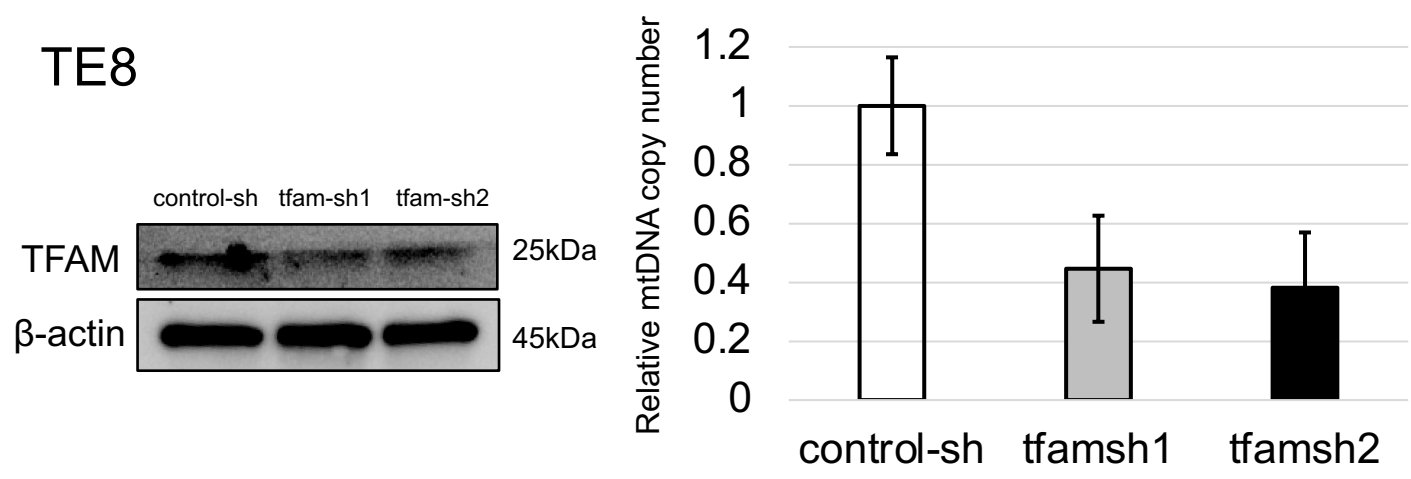

B

TE11

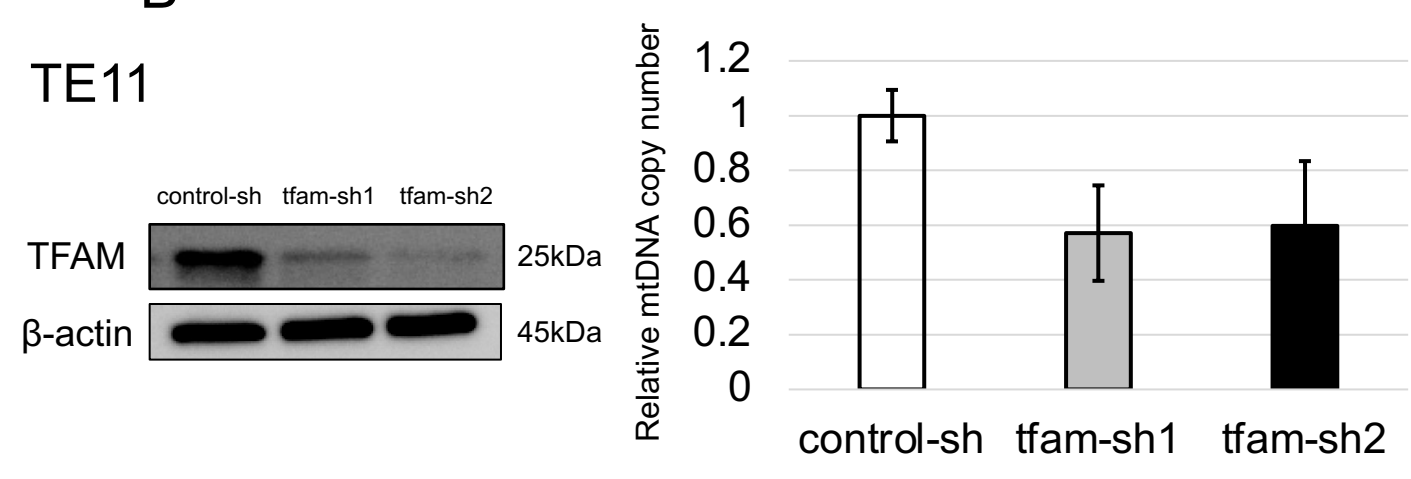

C

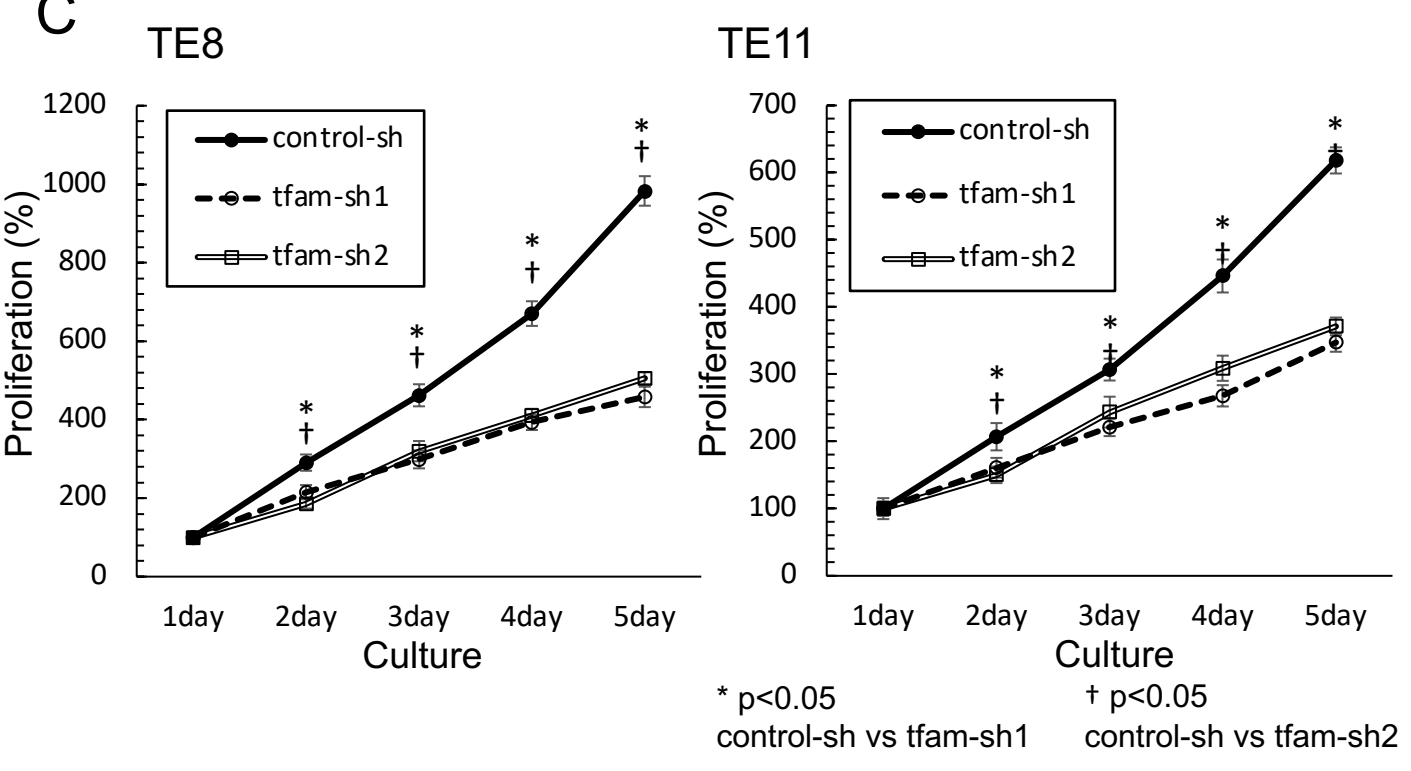

Supplement: Supplementary file 1 — Additional file 1: Figure S1. The association between TFAM knockdown and mtDNA copy number. A TFAM protein expression was lower in tfam-sh than control-sh TE8 cells. tfam-sh1 and tfam-sh2 TE8 cells had an approximately 60% decrease in mtDNA copy number compared with control-sh TE8 cells. B: tfam-sh cells had lower protein expression levels of TFAM than control-sh cells. The mtDNA copy number in tfam-sh1 and tfam-sh2 TE11 cells was approximately 40% lower than that in control-sh TE11 cells. C: The proliferation rates of tfam-sh1 and tfam-sh2 cells were significantly lower than that of control-sh cells. [file 12967_2022_3594_MOESM1_ESM.pdf]

Supplemental Figure. 2

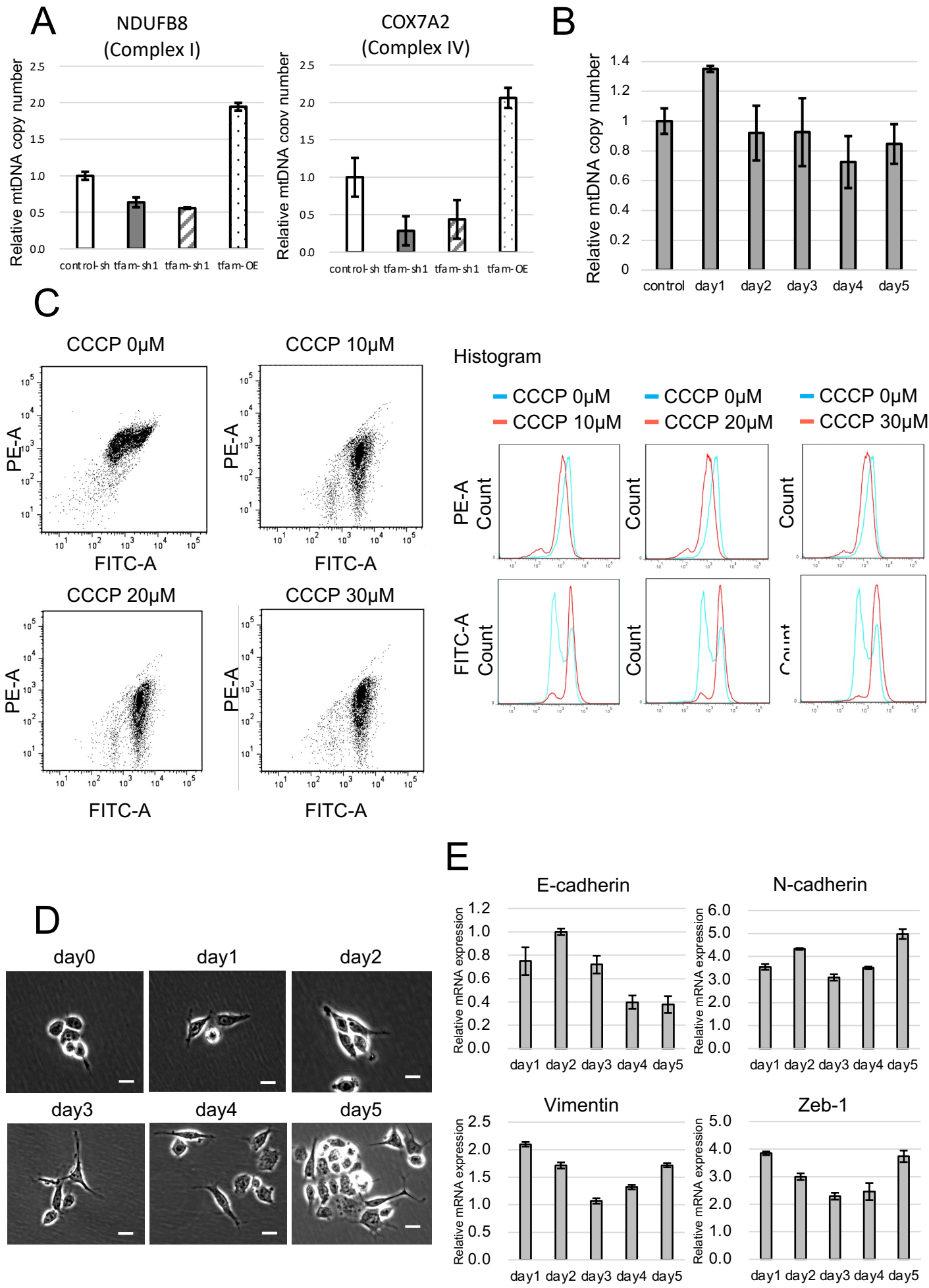

Supplement: Supplementary file 2 — Additional file 2: Figure S2. Mitochondrial membrane potential and epithelial–mesenchymal transition. A The complex I and IV mRNA expression levels was lower in mtDNA-depleted ESCC and was higher in mtDNA-increased ESCC. B The ESCC cells had no difference in mtDNA copy number under 40 μM CCCP exposure. C 30 µM CCCP induced to most decrease in mitochondrial membrane potential. D TE8 under 30 µM CCCP exposure showed spindle cell transformation, depending on exposure time (day1-5). Scale bars, 20 µM. E The relative mRNA expression level of E-cadherin in ESCC cells which cultured with CCCP was significantly decreased at day5 compared with control cells in TE8. The relative mRNA expression level of N-cadherin, vimentin, and zeb-1 in its cells was significantly increased compared with control cells. [file 12967_2022_3594_MOESM2_ESM.pdf]

Supplemental Figure. 3

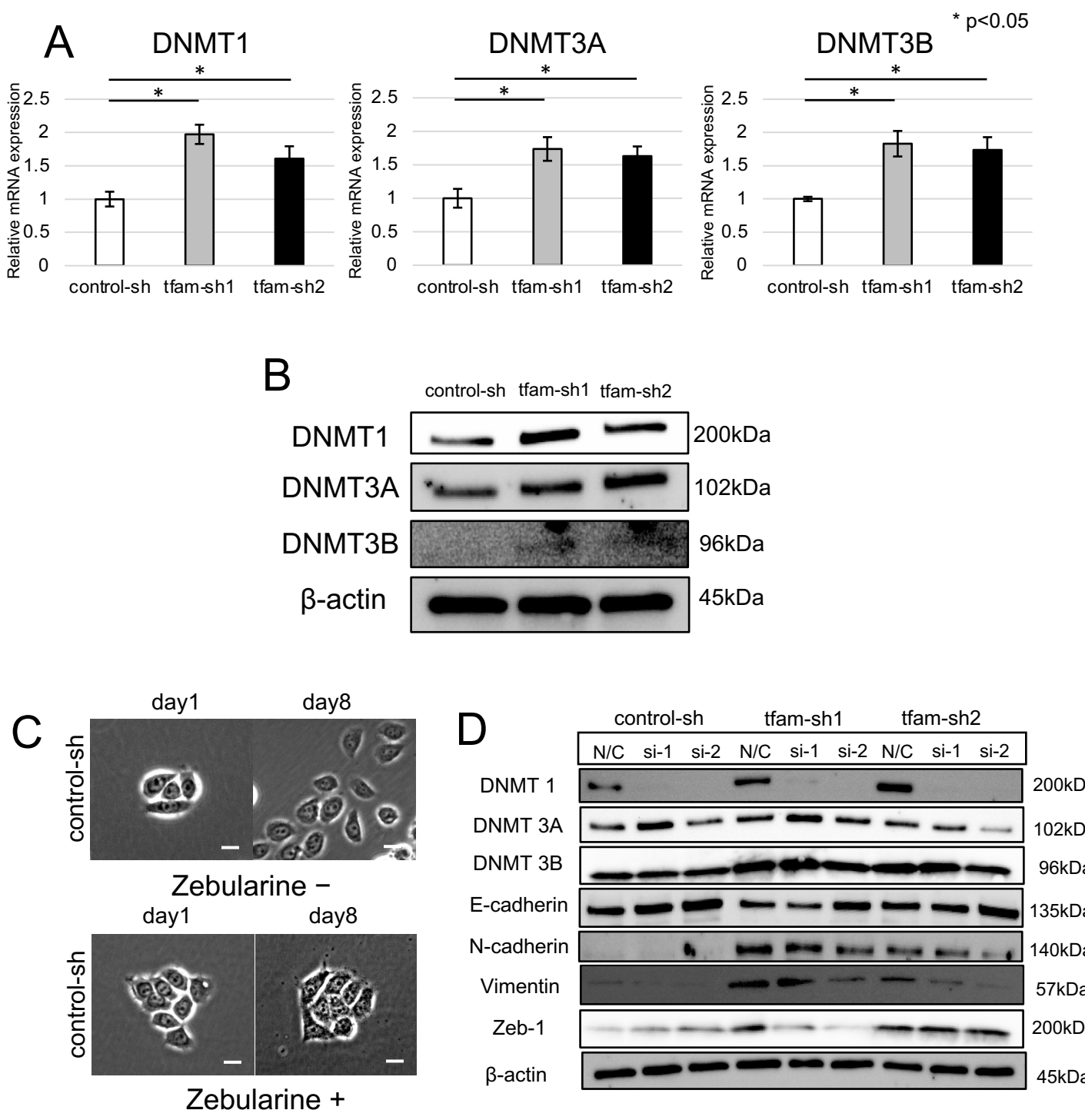

Supplement: Supplementary file 3 — Additional file 3: Figure S3. Relationship of mtDNA and DNA methylation. A The mtDNA-depleted of TE8 cells had appropriately double higher the mRNA expression level of DNMT-1, DNMT-3A and DNMT-3B than control cell lines. B A mtDNA-depleted of TE8 cells had higher protein expression levels of DNMT-1 and DNMT-3A compared with control cells. C The control cell morphology with DNMT inhibitor remained nonspindle and unchanged. Scale bars, 20 µM. D The DNMT1 protein level in control cells and mtDNA-depleted cells were lower expression by DNMT1 knockdown, and DNMT3A and 3B protein levels had little change in DNMT1 knockdown cell. Also, the protein level of E-cadherin was higher in mtDNA-depleted cells of DNMT1 knockdown. The N-cadherin and vimentin protein levels by DNMT1 knockdown had lower expression in mtDNA-depleted ESCC cells. [file 12967_2022_3594_MOESM3_ESM.pdf]

Supplemental Figure. 4

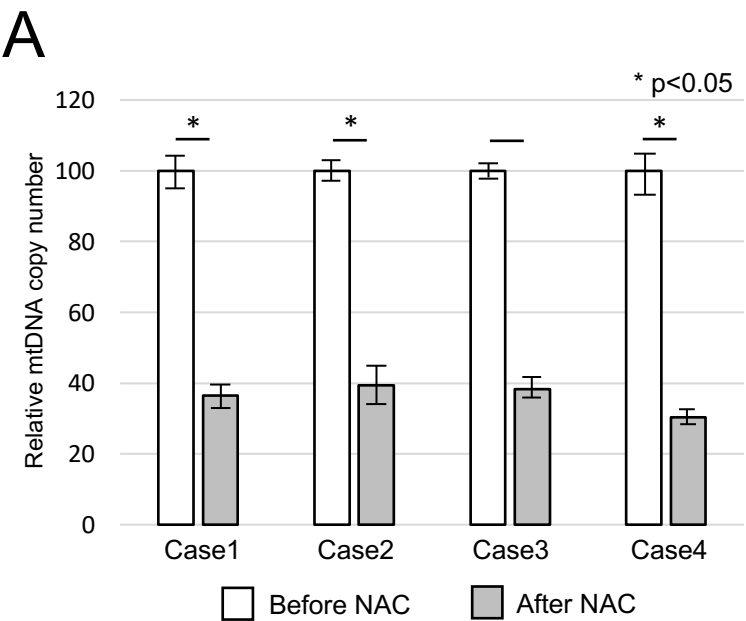

Supplement: Supplementary file 4 — Additional file 4: Figure S4. The change in mtDNA copy number after NAC. A The mtDNA copy number of the ESCC patients after NAC was about 40% lower than this patients before NAC. [file 12967_2022_3594_MOESM4_ESM.pdf]
