# Supplementary material for: Low mitochondrial DNA copy number induces chemotherapy resistance via epithelial-mesenchymal transition by DNA methylation in esophageal squamous cancer cells
Source: J Transl Med. 2022 Aug 29;20:383. doi: 10.1186/s12967-022-03594-2 (PMC9422107; doi:10.1186/s12967-022-03594-2)
Supplement: Supplementary file 5 — Additional file 5: Table S1. Primer used in this study. [file 12967_2022_3594_MOESM5_ESM.docx]

Table S1

Primer used in this study

| Target | Sequence |
| --- | --- |
| MTCO1 | Forward;5’-TGATCTGCTGCAGTGCTCTGA-3’  Reverse; 5’-TCAGGCCACCTACGGTGAA-3’ |
| COX IV | Forward;5’-GAAAGTGTTGTGAAGAGCGAAGAC-3’  Reverse;5’-GTGGTCACGCCGATCCAT-3’ |
| NDUFB8 (Complex I) | Forward;5-CATGGGGTATGGCGACTACC-3′  Reverse;5’-CGGTTCACCCCAGTTCAACC-3’ |
| COX7A2 (Complex IV) | Forward;5’-AGATTGGGCAGAGGACGATAA-3’  Reverse;5’-CCCACCCTTTAGATACAGTGGAA-3’ |
| E-cadherin (CDH1) | Forward;5’-GAGAAACAGGATGGCTGAAGG-3’  Reverse;5’-TGAGGATGGTGTAAGCGATGG-3’ |
| N-cadherin (CDH2) | Forward;5’-GACAATGCCCCTCAAGTGTT-3’  Reverse;5’-CCATTAAGCCGAGTGATGGT-3’ |
| Vimentin | Forward;5’-AGCTAACCAACGACAAAGCC-3’  Reverse;5’-TCCACTTTGCGTTCAAGGTC-3’ |
| Zeb-1 | Forward;5’-GGGCACAGGGTACAGGGAGA-3’  Reverse;5’-AATTGAGGGGCGAGGGAAAA-3’ |
| DNMT1 | Forward;5’-CCATCAGGCATTCTACCA-3’  Reverse;5’-CGTTCTCCTTGTCTTCTCT-3’ |
| DNMT3A | Forward;5’-TATTGATGAGCGCACAAGAGAGC-3’  Reverse;5’-GGGTGTTCCAGGGTAACATTGAG-3’ |
| DNMT3B | Forward;5’-TACACAGACGTGTCCAACATGGGC-3’  Reverse;5’-GGATGCCTTCAGGAATCACACCTC-3’ |
| GAPDH | Forward;5’-CGACTTCAACAGCGACACTCAC -3’  Reverse;5’-CCCTGTTGCTGTAGCCAAATTC -3’ |
